# Gut microbiota community and metabolic profiles in direct total cavopulmonary connection and Fontan circulation: a cross-sectional study in the single center

**DOI:** 10.3389/fmicb.2025.1539046

**Published:** 2025-03-14

**Authors:** Kaiyu Wang, Linjiang Han, Jianrui Ma, Yushen Fang, Yinru He, Xiaobing Liu, Shusheng Wen, Jian Zhuang, Haiyun Yuan

**Affiliations:** ^1^Department of Cardiovascular Surgery, Guangdong Cardiovascular Institute, Guangdong Provincial People’s Hospital, Guangdong Academy of Medical Sciences, Guangzhou, China; ^2^Guangdong Provincial Key Laboratory of South China Structural Heart Disease, Guangzhou, China; ^3^The Second School of Clinical Medicine, Southern Medical University, Guangzhou, China

**Keywords:** direct total cavopulmonary connection, Fontan procedure, microbiome, metabolome, congenital heart surgery

## Abstract

**Background:**

This study aimed to evaluate the impact of the direct total cavopulmonary connection (dTCPC) procedure on the gut microbiome and metabolome. It also sought to elucidate the features of the gut microbiota community and metabolic profiles in Fontan circulation.

**Methods:**

We randomly recruited 45 participants above 14 years old undergoing Fontan procedure by typical extracardiac conduit (TCPC group, *n* = 15), direct total cavopulmonary connection (dTCPC group, *n* = 16) procedure and healthy control (control group, *n* = 14) in our institution between May 2023 and October 2023. 16S rRNA amplicon sequencing and untargeted metabolites measurement were performed on their fecal sample.

**Results:**

The four alpha diversity indexes showed no statistical significance between the dTCPC and TCPC groups (*p* > 0.05). Orthogonal partial least squares discriminant analysis (OPLS-DA) followed by permutation testing indicated an overfitting effect in the model between the dTCPC and TCPC groups. We observed significant differences in the Chao1 index (*p* = 0.0236), the ACE index (*p* = 0.0236), and the unweighted beta diversity (*p* = 0.0099) between the Fontan group and healthy control group. Strains of *Fusobacterium* were significantly overrepresented in the Fontan group [with linear discriminant analysis (LDA) scores exceeding (log10) >3]. Functional enrichment analysis revealed a significant overrepresentation of several metabolic pathways. These pathways predominantly included those related to amino acid metabolism, such as histidine metabolism, glycine, serine, and threonine metabolism, and cysteine and methionine metabolism. Additionally, the biosynthesis of unsaturated fatty acids was also notably enriched.

**Conclusion:**

The dTCPC procedure demonstrated similar gut microbiota composition and metabolic profiles to the traditional ECC procedure in Fontan patients. Notably, the increased abundance of *Fusobacterium*, reduced microbial biodiversity, and altered metabolic profiles of amino acids and unsaturated fatty acids in the alimentary canal may serve as distinctive characteristics of patients who have undergone Fontan circulation. These findings provide valuable insights into the long-term physiological consequences of Fontan procedure and may inform future clinical management strategies.

## Introduction

The total cavopulmonary connection (TCPC) has become the predominant surgical intervention for functional single ventricle (FSV) malformation due to its favorable long-term outcomes (Dennis et al., 2018). However, due to the TCPC structure, Fontan physiology is characterized by high-pressure, non-pulsatile flow in the inferior vena cava, leading to blood stagnation and reflux within the digestive system (Emamaullee et al., 2020). This condition often results in Fontan-associated liver disease (FALD), protein-losing enteropathy (PLE), and malnutrition. A new modified TCPC procedure named direct total cavopulmonary connection (dTCPC) was developed by our research team and demonstrated favorable midterm results in eligible participants (Liu et al., 2020). However, it is unclear how our surgical approach affects the digestive system of patients with FSV compared to the typical extracardiac conduit (ECC) procedure.

Recent studies have highlighted the role of gut dysbiosis in cardiovascular diseases (Witkowski et al., 2020). Alterations in gut bacteria and metabolites strongly correlate with the severity of coronary artery disease (Liu et al., 2019; Talmor-Barkan et al., 2022; Dong et al., 2023). Moreover, gut microbe-derived metabolites, including trimethylamine-N-oxide (TMAO) and phenylacetyl glutamine (PAGln), have been shown to play a role in atherosclerosis and thrombosis (Witkowski et al., 2020; Dong et al., 2023). Existing research on Fontan circulation has indicated that decreased diversity in the gut microbiota may be associated with the severity of PLE (Go et al., 2024). However, to the best of our knowledge, the alterations in the microbiome and metabolome of the alimentary canal in patients who have undergone the dTCPC procedure and Fontan circulation have not been thoroughly explored.

To address this gap in knowledge, we conducted non-targeted metabolomics and 16S rRNA sequencing on fecal samples from two groups of participants and compared their gut microbial community and fecal metabolic profiles.

## Methods

### Ethics statement

This study was approved by the Ethics Committee of Guangdong Provincial People’s Hospital, in accordance with the ethical standards of the 1964 Declaration of Helsinki and its later amendments (Approval KY2023-543-01). Written informed consent was obtained from all participants.

### Study design

This was a single-center follow-up study. For the dTCPC cohort, no new patients were included since the last report (Liu et al., 2020). In general, all patients who underwent the dTCPC procedure at our institution without Fontan failure (*n* = 23) were contacted. A total of 16 patients were willing to cooperate and return to our institution for a systematic examination between May 2023 and October 2023. For the ECC cohort in the Fontan group, a total of 268 patients were included from October 2009 to August 2021. To minimize potential selection bias, a propensity score-matching (PSM) analysis was performed to match the dTCPC candidates in the ECC cohort (1:1) according to baseline variables, including gender, age at Fontan, weight, height, and BSA. A total of 15 eligible patients who underwent ECC were recalled to the hospital for examination, except for one patient who could not attend due to personal reasons. In total, 45 participants were enrolled in this study, including healthy controls (control group, *n* = 14), patients who underwent ECC (TCPC group, *n* = 15), and patients who underwent dTCPC (dTCPC group, *n* = 16). The operative techniques of dTCPC have been previously published (Liu et al., 2020). The exclusion criteria included the following: (i) diagnosis of any extracardiac medical condition that could affect metabolism, nutritional status, or physical health; and (ii) prior use of probiotics, antibiotics, or immunosuppressants, which could potentially affect gut microbial composition. The fecal samples were collected on the first morning after admission to the hospital and immediately frozen at −80°C until analysis.

### Untargeted metabolites measurement of the feces

After thawing the samples at 4°C, 25 mg was weighed and placed into 800 μL of an extraction solution (methanol: acetonitrile: water = 2:2:1, pre-cooled to −20°C), along with 10 μL of an internal standard mixture and a steel ball. The mixture was homogenized using a tissue grinder (50 Hz, 5 min), followed by ultrasonic bath treatment in 4°C water for 10 min After incubation at 20°C for 1 h, the samples were centrifuged at 25,000 g for 15 min at 4°C. The supernatant (approximately 600 μL) was collected and dried using a freeze vacuum concentrator. Then, the solid matter was redissolved in 600 μL of a complex solution (methanol: H_2_O = 1:9). This solution was then vortexed, sonicated, and centrifuged under the same conditions as above. The final supernatant was transferred to a vial for analysis. Quality control (QC) samples were prepared by taking 50 μL of the supernatant from each sample to assess the repeatability and stability of the LC-MS analysis. Metabolites were detected and separated using a Waters UPLC I-Class Plus system (Waters, United States) coupled with a Q Exactive high-resolution mass spectrometer (Thermo Fisher Scientific, United States). The separation was performed using a Waters ACQUITY UPLC BEH C18 column (1.7 μm, 2.1 × 100 mm), with mobile phases containing water with 0.1% formic acid (phase A) and acetonitrile (phase B). The metabolites were separated at 0.35 mL/min using the following gradient conditions: 0–1 min, 2% B; 1–9 min, 2–98% B; 9–12 min, 98% B; 12–12.1 min, 98% B to 2% B; and 12.1–15 min, 2% B. Primary and secondary mass spectrometry data were acquired using a Q Exactive spectrometer (Thermo Fisher Scientific, United States). The full scan range was 70–1,050 *m*/*z* with a resolution of 70,000. The top three precursors were selected for subsequent MS/MS fragmentation. The stepped normalized collision energy was set to 20, 40, and 60 eV. The mass spectrometry data were processed using Compound Discoverer 3.3 (Thermo Fisher Scientific, United States). After identifying the metabolite structures and pre-processing the data, the data were qualitatively evaluated (Zeng et al., 2017).

### 16S rRNA amplicon sequencing

Genomic DNA and fusion primers were PCR-amplified and purified using Agencourt Ampure XP beads. The products were then eluted, labeled, and prepared for library construction. The Agilent 2100 Bioanalyzer system assessed the fragment size and concentration, and the qualified libraries were sequenced. Clean data were obtained by extracting target regions using Cutadapt V2.6, followed by specific filtering steps. Denoising with DADA2 in QIIME2 resulted in 100% similar ASVs, from which a feature table was generated. The ASV representative sequences were aligned with the database for species annotation using the RDP Classifier (1.9.1) at a confidence threshold of 0.6.

### Statistical analysis

Given the sample size of this study, the median (IQR) was used to describe continuous variables, and the Mann–Whitney *U* test was applied to compare these variables between the two groups. Categorical variables were expressed as frequencies with percentages, and comparisons were performed using a chi-squared test or Fisher’s exact test, as appropriate. For PSA, the nearest neighbor matching algorithm was applied, with a caliper width of 0.2 of the standard deviation of the logit of the propensity score, resulting in 16 well-matched pairs. A two-tailed *p*-value of <0.05 was considered statistically significant. Analyses were conducted using SPSS 26.0 and R 4.2.1. For 16S rRNA gut microbiome analysis, alpha diversity (ACE, Chao1, Shannon, and Simpson) was assessed using QIIME2, while beta diversity and PCoA were analyzed using the Bray–Curtis index and unweighted UniFrac distances in R. Linear discriminant analysis (LDA) effect sizes identified dominant bacterial differences, and fecal microbiota functions were predicted using the MetaCyc database. For non-targeted metabolomics, orthogonal partial least squares discriminant analysis (OPLS-DA) was used to identify relationships between the metabolites and sample categories, with significant metabolites selected based on VIP >1 and *p* < 0.05. Pathway enrichment analysis was performed using the KEGG database.

## Results

### Baseline characteristics

The detailed demographic characteristics of the TCPC and dTCPC groups have been published in a previous article by our group (Wang et al., 2024). In brief, the mean follow-up duration was 144.00 (43.70) months for the TCPC group and 174.25 (63.06) months for the dTCPC group. None of the participants experienced severe complications, such as cardiac dysfunction, FALD, PLE, or thromboembolic events. The cardiac output (CO) values showed no significant variation between the two groups [5.28 (3.20) vs. 5.40 (2.85), *p* > 0.05].

### Comparison of the intestinal flora between dTCPC and TCPC

**Figure 1:**
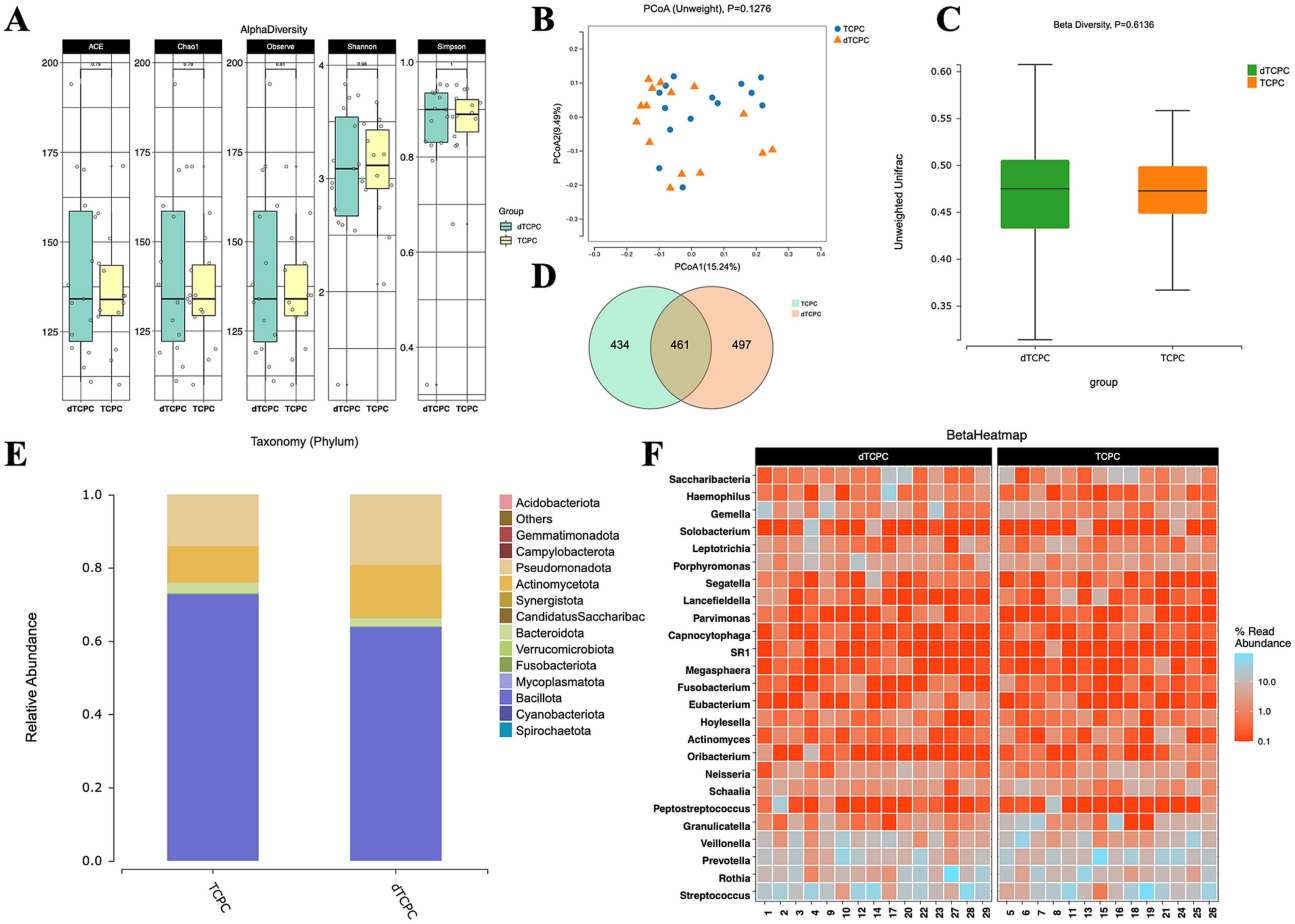
Fecal microbiome diversity and structure analysis between the dTCPC and TCPC groups. **(A)** The alpha diversity indexes of the fecal differences between the dTCPC and TCPC groups were estimated using the Shannon, Simpson, ACE, and Chao1 indexes. The unweighted PCoA plots **(B)** and unweighted beta diversity **(C)** did not reveal a separation between the two groups in the fecal samples. **(D)** Venn diagrams show the existence of ASVs with a relative abundance >0.1% in each group. **(E)** Fecal species stacking diagram at the phylum level. **(F)** Profile of the top 25 most abundant fecal genera at the genus level.

The alpha diversity metrics, including the Chao1 index (*p* = 0.74), ACE index (*p* = 0.73), Simpson index (*p* = 0.95), and Shannon index (*p* = 0.98), showed no significant differences between the groups (Figure 1A). Similarly, the unweighted PCoA plots (Figure 1B) demonstrated no distinct separation based on the first two principal coordinates, which aligns with the unweighted beta diversity statistics (*p* = 0.6136) (Figure 1C). Venn diagrams of the ASVs revealed that approximately one-third of the ASVs were shared between the groups (Figure 1D). The relative abundance of dominant taxa at the phylum level (Figure 1E) showed that Bacillota was the predominant phylum in both groups, constituting 63.81% in the dTCPC group and 72.83% in the TCPC group (Supplementary Table S1). The top 25 genera by abundance are presented in Figure 1F. These findings suggest that gut microbiota diversity was minimally affected by the dTCPC procedure.

### Comparison of the intestinal non-targeted metabolic profiles of dTCPC and TCPC

The orthogonal partial least squares discriminant analysis (OPLS-DA), followed by permutation testing, indicated model overfitting, suggesting minimal differences between the groups (Supplementary Figures S1A,B). Overall, untargeted metabolomics demonstrated that the fecal metabolic profiles of the patients who underwent dTCPC and TCPC were largely similar.

### Overview of the intestinal flora between the patients who underwent Fontan and healthy individuals

**Figure 2:**
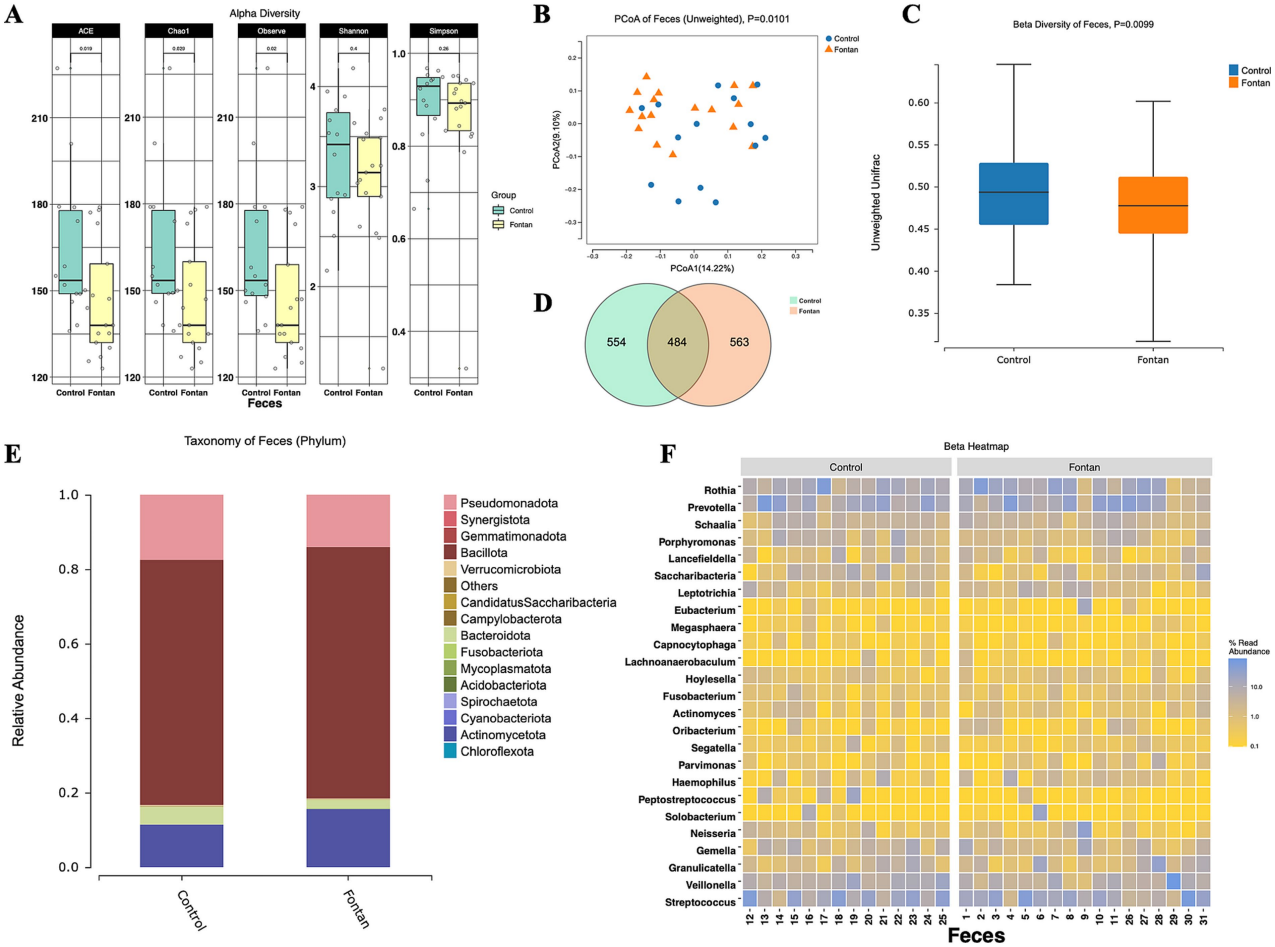
Fecal microbiome diversity and structure analysis between the Fontan and control groups. **(A)** The alpha diversity indexes of the fecal differences between the Fontan and Control groups were estimated using the Shannon, Simpson, ACE, and Chao1 indexes. The unweighted PCoA plots **(B)** and unweighted beta diversity **(C)** revealed a separation between the two groups in the fecal samples. **(D)** Venn diagrams show the existence of ASVs with a relative abundance >0.1% in each group. **(E)** Fecal species stacking diagram at the phylum level. **(F)** Profile of the top 25 most abundant fecal genera at the genus level.

To further elucidate the impact of Fontan surgery on the gut microbiota, we stratified the cohort into two subgroups based on age (over 18 years) and included healthy adults as controls. Notable differences in the alpha diversity were observed in the Chao1 index (*p* = 0.0236) and ACE index (*p* = 0.0236) between the Fontan and control groups (Figure 2A). The unweighted PCoA plots demonstrated a distinct separation between the groups based on the first two principal coordinates (Figure 2B), which aligns with the unweighted beta diversity metrics (*p* = 0.0099) (Figure 2C). Venn diagrams (Figure 2D) showed that approximately one-third of the ASVs were common to both groups. The analysis of dominant taxa at the phylum level (Figure 2E) showed that Bacillota was the most abundant phylum, with a relative abundance of 67.54% in the Fontan group and 65.83% in the control group (Supplementary Table S2). The top 25 abundant genera at the genus level are depicted in Figure 2F. These findings suggest that Fontan circulation may influence gut microbiota diversity to some extent.

### Alterations in the composition of the fecal microflora of the Fontan patients

**Figure 3:**
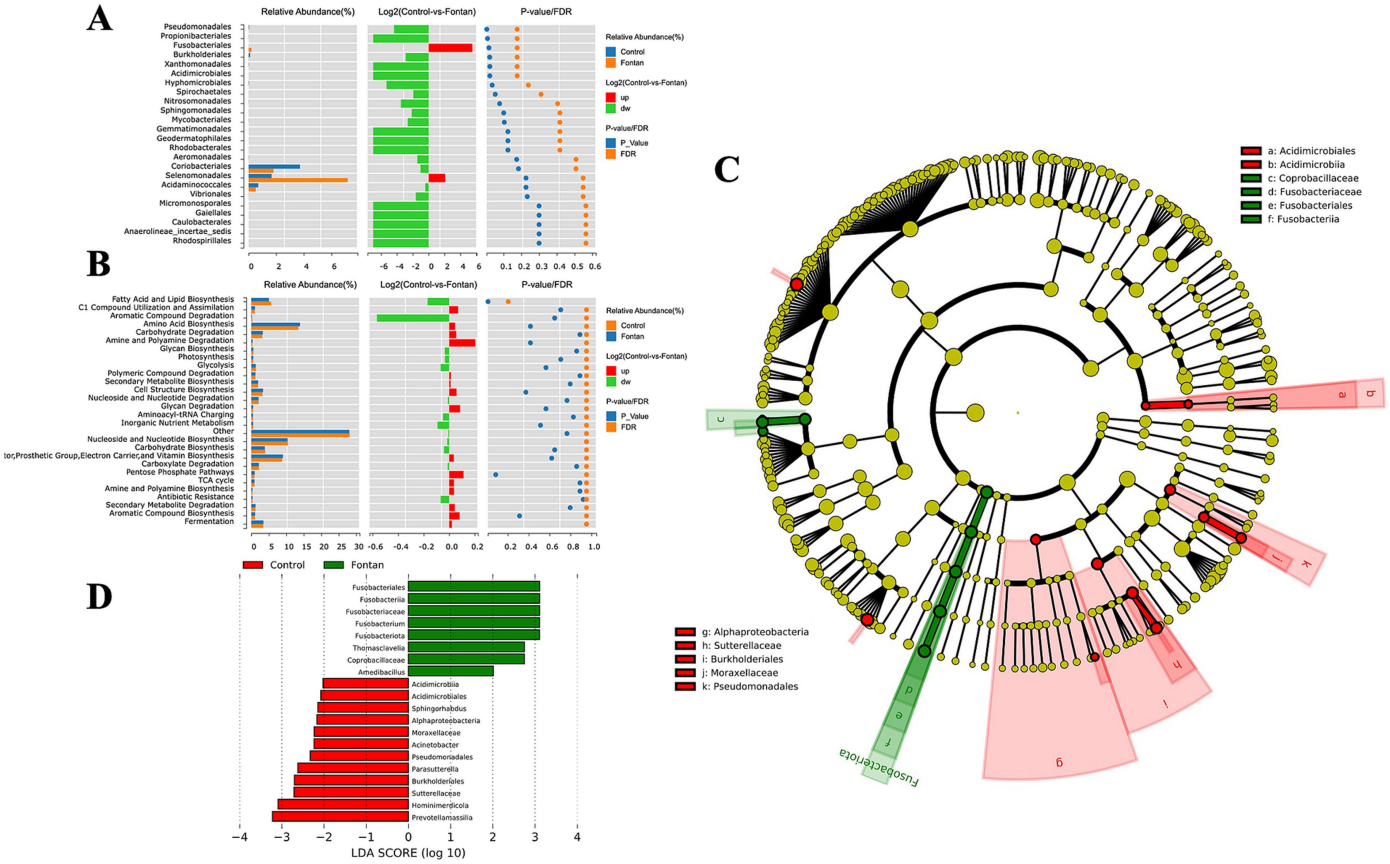
Fecal microbiome variance analysis between the Fontan and control groups. **(A)** Composition analysis of differential microbial flora. **(B)** Enrichment analysis of the MetaCyc pathway in different microbiota. **(C,D)** Show LEfSe analysis and a cladogram used to distinguish between the two groups.

By adjusting the level to FDR ≤0.2, only five taxa were selected at the order level, namely Pseudomonadales, Propionibacteriales, Fusobacteriales, Burkholderiales, and Xanthomonadales (Figure 3A). The enrichment analysis using the MetaCyc database revealed that the differential microbial components were primarily enriched in fatty acid and lipid biosynthesis pathways (FDR = 0.2040) (Figure 3B). Given the unsatisfactory results from this analysis, we performed a LEfSe analysis to generate a cladogram that identifies specific bacteria associated with the Fontan group (Figure 3C). *Fusobacterium* was significantly overrepresented in the Fontan group, whereas *Prevotellamassilia* and *Hominimerdicola* were the most abundant in the control group (Figure 3D). Overall, these data suggested a reduced microbial abundance in the Fontan group, with differentially abundant microbiota effectively distinguishing between healthy individuals and patients with Fontan circulation.

### Overview of the fecal metabolism in the Fontan patients

**Figure 4:**
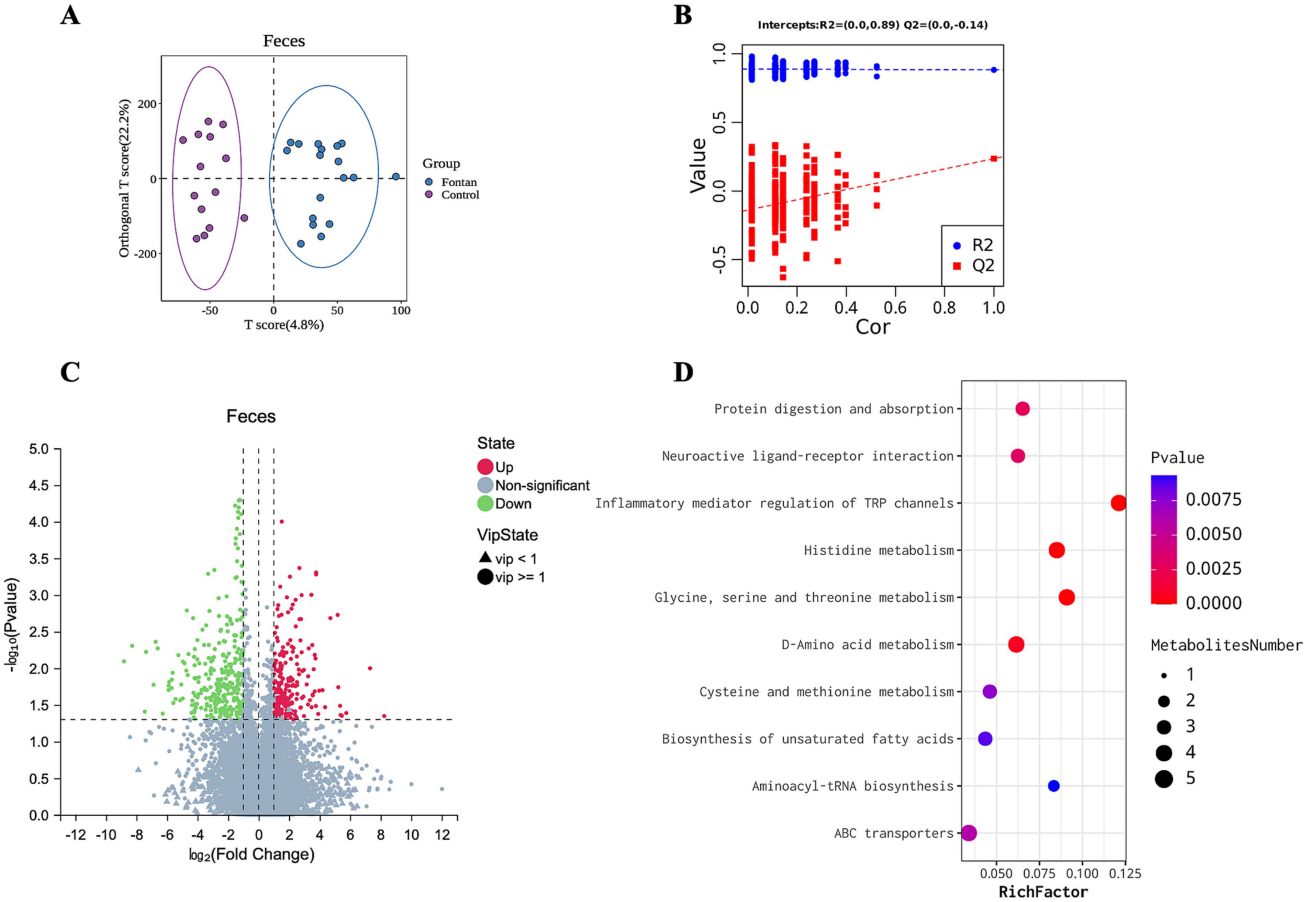
Discrepant fecal metabolic profile between the Fontan and control groups. **(A)** OPLS-DA and **(B)** permutation testing showed no overfitting effect in the model. **(C)** Metabolite volcano map. **(D)** KEGG pathway enrichment analysis of the metabolites.

The OPLS-DA score scatter plots of the two samples are shown in Figures 4A,B, which show that the model had no fitting effect in the permutation test. After thresholding the metabolites with a VIP value >1 and a Wilcoxon rank-sum *p*-value <0.05, a total of 544 metabolic features that significantly differed in abundance between the groups were identified, with a fold change in concentration ≥2 (Figure 4C) (Supplementary material). The functional enrichment analysis focused on histidine metabolism, glycine, serine, and threonine metabolism, cysteine and methionine metabolism, D-amino acid metabolism, and the biosynthesis of unsaturated fatty acids, among others (Figure 4D).

## Discussion

Our analysis of the fecal 16S rRNA and metabolomics revealed the following: (1) dTCPC and traditional TCPC surgeries had comparable gut microbiota biodiversity and composition in the long-term prognosis; (2) the gut microbiota metabolic profiles were also similar between the two groups; (3) compared to the healthy controls, the Fontan patients exhibited an increased proportion of *Fusobacterium* and reduced biodiversity; and (4) the metabolic profiles of amino acids and unsaturated fatty acids were altered in Fontan patients.

The dTCPC procedure theoretically offers a considerable advantage in reducing postoperative thromboembolic events due to the exclusion of additional prosthetic materials, distinguishing it from other Fontan procedures. Postoperatively, the patients were prescribed aspirin at 3–5 mg/kg/day for a period of 3–6 months. Our preliminary retrospective study showed no significant differences in long-term survival rates or serious complications between the dTCPC and traditional ECC cohorts (Liu et al., 2020). Our study provides a novel perspective for evaluating the quality of life post-dTCPC surgery through gut microbiota and metabolite analysis. Notably, the species richness index of the gut microbiota and the OPLS-DA of the metabolites did not effectively distinguish between the groups. Thus, our findings suggest that the dTCPC procedure may be a viable and advantageous option for selected patients compared to the traditional TCPC method.

A multicenter case-control study also identified the dysbiosis of the gut microbiota in patients with protein-losing enteropathy after the Fontan procedure using 16S rRNA sequencing. LEfSe demonstrated differences in the relative abundance of *Bifidobacterium*, *Granulicatella* spp., and *Ruminococcus torques* between patients in the active and remissive stages of PLE (Go et al., 2024). However, this study did not include individuals with normal circulation as controls. Our findings revealed a significant increase in *Fusobacterium* abundance in the Fontan group, indicating its potential as a biomarker to distinguish between the groups. It is noteworthy that *Fusobacterium*, typically present in the oral cavity and rarely found in the lower gastrointestinal tract of healthy individuals, is enriched in colorectal cancer (CRC) tumors. High levels of intratumoral *Fusobacterium* are associated with recurrence, metastases, and poorer prognosis (Zepeda-Rivera et al., 2024). Although the disease types differ, the similar pathological features and treatment responses of PLE and inflammatory bowel disease (IBD) (Kewcharoen et al., 2020; Rodríguez de Santiago et al., 2020) warrant caution regarding the elevated levels of *Fusobacterium* observed.

Previous research has documented significant changes in plasma metabolic components in patients with Fontan circulation. For instance, Adam et al. observed that Fontan circulation is linked to reduced cholesterol levels, with lower high-density lipoprotein cholesterol being associated with adverse clinical outcomes (Lubert et al., 2021). They speculated that these observations could be attributed to liver dysfunction secondary to FALD, given the liver’s essential role in lipid metabolism and the common occurrence of lipoprotein deficiencies in chronic liver disease (McIntyre, 1978). A cross-sectional study identified notable differences in the metabolomes of phosphatidylcholine, sphingomyelin, and acylcarnitine between Fontan patients and healthy controls (Michel et al., 2020b). The authors also revealed alterations in plasma amino acid metabolomics (Michel et al., 2020a). A metabolomic study highlighted that the concentrations of organic acids (2-oxoglutaric acid and cis-aconitic acid) related to the tricarboxylic acid (TCA) cycle were significantly higher in Fontan patients compared to healthy controls, which was speculated to be the cause of the manifestation of compensatory increase in cardiac output and disruption of energy metabolism in the liver (Motoki et al., 2021). Several studies have also suggested that combining metabolomic data with imaging indicators could enhance the prediction of FALD risk in post-Fontan patients, as conventional liver function tests often fail to detect issues until the disease reaches advanced stages (Daniels et al., 2017; Emamaullee et al., 2020; Motoki et al., 2021).

In contrast to the above studies, the current study employed non-targeted metabolomics analysis on the fecal samples of the Fontan patients, providing a more immediate and comprehensive insight into the metabolic state of the gut microbiota in Fontan circulation. Existing research has shown that the methionine metabolite homocysteine in feces is linked to atherothrombosis and may cause the development of myocardial infarction (Dong et al., 2023). In addition, fecal phenylalanine and tyrosine levels were also significantly correlated with an increased risk of CAD (Dong et al., 2023). Our results similarly revealed that Fontan circulation leads to enhanced methionine and cysteine metabolism in the intestinal environment. However, due to limitations in our sample size and the incidence of Fontan complications within our study cohort, we were unable to draw causal inferences regarding the clinical significance of all observed amino acid metabolic alterations. Consequently, stratifying the post-Fontan patients based on metabolomic profiles for risk assessment was not feasible within the scope of this study. Future research should aim to incorporate a larger cohort to address these limitations.

Our study has several limitations. First, the sample size was relatively small due to the inherently limited dTCPC cohort and the high surgical expertise required for these procedures, resulting in infrequent performance. Therefore, we interpreted our results with caution. In addition, the previously published results from our research team have indirectly demonstrated the reliability of the data in the present study. Our previous research indicated that there were no significant differences in long-term freedom from death or transplantation and freedom from Fontan failure between dTCPC and ECC groups (Liu et al., 2020). However, for patients who have not experienced Fontan circulation failure, long-term survival quality following dTCPC surgery remains unsatisfactory (Wang et al., 2024). Second, the randomly selected participants did not experience any significant adverse events, including Fontan-related gastrointestinal complications. In addition, liver MRI scans and colorectal endoscopies were not performed on these patients, preventing us from directly correlating clinical outcomes with omics results or identifying relevant biomarkers for precise risk stratification. In the future, our team will expand the cohort by including more patients and improving imaging assessments. Moreover, the use of probiotics to regulate gut microbiota homeostasis associated with Fontan circulation may become a promising direction for further investigation.

## Conclusion

Our study revealed that patients who underwent the dTCPC surgery exhibited long-term gut microbiota biodiversity, composition, and metabolic profiles comparable to those who received the traditional ECC surgery. When compared to the healthy controls, the Fontan patients, regardless of surgical approach, demonstrated some alterations in their gut microbiome. These patients exhibited an increased abundance of *Fusobacterium* and reduced overall microbial biodiversity. Moreover, the metabolomic analysis revealed significant perturbations in the amino acid and unsaturated fatty acid metabolic pathways. These observed changes in the gut microbiota and metabolome may serve as potential biomarkers for assessing long-term outcomes in patients with Fontan circulation.

## Data Availability

The datasets presented in this study can be found in online repositories. The names of the repository/repositories and accession number(s) can be found in the article/Supplementary material.
