# Supplementary material for: Gut microbiota community and metabolic profiles in direct total cavopulmonary connection and Fontan circulation: a cross-sectional study in the single center
Source: Front Microbiol. 2025 Mar 14;16:1539046. doi: 10.3389/fmicb.2025.1539046 (PMC11952763; doi:10.3389/fmicb.2025.1539046)
Supplement: Supplementary file 1 [file Table_1.docx]

Table S1

Relative abundance of species of dTCPC and TCPC

|  | TCPC | dTCPC |
| --- | --- | --- |
| Spirochaetota | 0 | 3.42E-05 |
| Cyanobacteriota | 3.37E-05 | 1.83E-04 |
| Bacillota | 0.728310393 | 0.638058472 |
| Mycoplasmatota | 1.06E-05 | 2.64E-05 |
| Chloroflexota | 0 | 0 |
| Fusobacteriota | 0.001813938 | 0.001636486 |
| Verrucomicrobiota | 0.002653461 | 1.62E-04 |
| Bacteroidota | 0.025971974 | 0.022446119 |
| CandidatusSaccharibacteria | 3.44E-04 | 7.16E-04 |
| Synergistota | 4.61E-05 | 5.08E-04 |
| Actinomycetota | 0.101058722 | 0.143799382 |
| Pseudomonadota | 0.139435407 | 0.192224129 |
| Campylobacterota | 2.36E-04 | 1.99E-04 |
| Gemmatimonadota | 3.55E-06 | 0 |
| Others | 7.28E-05 | 6.22E-06 |
| Acidobacteriota | 8.87E-06 | 0 |

Table S2

Relative abundance of species of Fontan and Control

|  | Control | Fontan |
| --- | --- | --- |
| Chloroflexota | 1.37E-05 | 0 |
| Actinomycetota | 0.114176713 | 0.15618719 |
| Cyanobacteriota | 1.49E-04 | 1.65E-04 |
| Spirochaetota | 4.78E-05 | 1.41E-05 |
| Acidobacteriota | 1.91E-04 | 7.04E-06 |
| Mycoplasmatota | 1.54E-05 | 2.39E-05 |
| Fusobacteriota | 5.30E-05 | 0.002133878 |
| Bacteroidota | 0.047470409 | 0.022530371 |
| Campylobacterota | 2.89E-04 | 2.08E-04 |
| CandidatusSaccharibacteria | 0.001317246 | 6.11E-04 |
| Others | 0.001103685 | 4.52E-04 |
| Verrucomicrobiota | 0.001975015 | 0.002104299 |
| Bacillota | 0.658305314 | 0.675380119 |
| Gemmatimonadota | 2.56E-05 | 2.82E-06 |
| Synergistota | 2.27E-04 | 1.23E-04 |
| Pseudomonadota | 0.174640192 | 0.140057044 |
