# Supplementary figures and images for: Gut microbiota community and metabolic profiles in direct total cavopulmonary connection and Fontan circulation: a cross-sectional study in the single center
Source: Front Microbiol. 2025 Mar 14;16:1539046. doi: 10.3389/fmicb.2025.1539046 (PMC11952763; doi:10.3389/fmicb.2025.1539046)

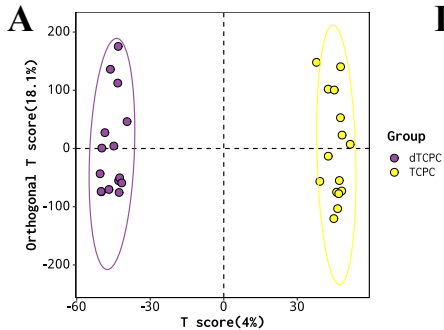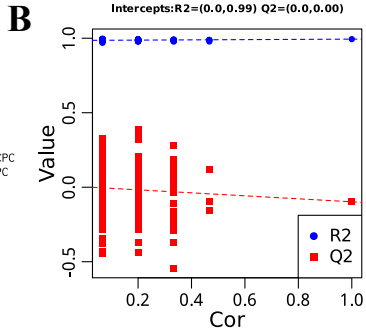

Supplement: Supplementary file 2 [file Data_Sheet_1.pdf]
